# Low Prevalence of Cysticercosis and *Trichinella* Infection in Pigs in Rural Cambodia

**DOI:** 10.3390/tropicalmed6020100

**Published:** 2021-06-11

**Authors:** Rebecca Söderberg, Johanna Frida Lindahl, Ellinor Henriksson, Kang Kroesna, Sokong Ly, Borin Sear, Fred Unger, Sothyra Tum, Hung Nguyen-Viet, Gunilla Ström Hallenberg

**Affiliations:** 1 Department of Clinical Sciences, Swedish University of Agricultural Sciences, 75007 Uppsala, Sweden; rebecca.soderberg@slu.se (R.S.); ellinorhenriksson@hotmail.com (E.H.); gunillastrom1@gmail.com (G.S.H.); 2 Animal and Human Health Program, International Livestock Research Institute, Hanoi 100 000, Vietnam; h.nguyen@cgiar.org; 3 Department of Medical Biochemistry and Microbiology, Uppsala University, 75236 Uppsala, Sweden; leesokong@gmail.com; 4 Faculty of Veterinary Medicine, Royal University of Agriculture, Phnom Penh 12201, Cambodia; kkroesna@rua.edu.kh (K.K.); borin_sear2007@yahoo.com (B.S.); F.Unger@cgiar.org (F.U.); 5 National Animal Health and Production Research Institute, General Directorate of Animal Health and Production, Phnom Penh 12350, Cambodia; sothyratum@gmail.com; 6 Public Health Agency Sweden, 17165 Stockholm, Sweden

**Keywords:** *Taenia solium*, *Taenia asiatica*, *Trichinella* spp., neglected tropical disease, food safety, parasitic disease, zoonoses

## Abstract

Cysticercosis and *Trichinella* spp. infection are parasitic zoonoses prevalent among pigs in Southeast Asia, where pork is the most important source of meat. In rural Cambodia, many pigs are raised extensively in family backyards, and information regarding the prevalence in rural small-scale pig production is very limited. This study was conducted in four provinces in north-eastern Cambodia to determine the seroprevalence of porcine cysticercosis and *Trichinella* spp. infection in rural villages, and to identify possible risk factors. Only households with less than 10 pigs above three months old were eligible. In total, 139 households participated, and 242 blood samples were collected. Farmers were interviewed about food and hygiene habits, disease knowledge and practices. The serum samples were analysed by ELISA to determine antigens to *Taenia* spp. cysticerci or antibodies to *Trichinella* spp. muscle larvae. Positivity among the pigs was 11.2% (95% CI 7.5–15.8) for *Taenia* spp. cysts and 2.5% (95% CI 0.9–5.4) for *Trichinella* spp. Cysticerci were more common in the province Preah Vihear (*p <* 0.001) than in the other provinces. Risk factors associated with porcine cysticercosis were management systems for the pigs and access to human faeces (*p* < 0.001). *Trichinella* spp. infection in pigs was more common in the province Ratanakiri (*p =* 0.001). The main risk factor associated with *Trichinella* spp. transmission was feeding pigs with food waste (*p =* 0.048). More men had heard about cysticercosis than women (*p =* 0.002), and men also consumed undercooked pork meat to a greater extent (*p =* 0.004). Although the present study is relatively small, several risk factors could be identified for porcine infection with *Taenia* spp. and *Trichinella* spp., which can be used to guide future interventions to improve both porcine and human health in these provinces.

## 1. Introduction

Cambodia is a lower middle-income country in Southeast Asia with a population of approximately 17 million inhabitants, a rapidly growing economy and decreasing poverty rates [1,2], but there is still a large proportion of the inhabitants, around 4.5 million, who remain near-poor and vulnerable to falling back into poverty if exposed to economic shock [1]. Approximately three quarters of the Cambodian population live in rural areas and around 90% of the poor live in the countryside [1,3].

Pork is the most important source of meat in this region [4] and in Cambodia, 80% of approximately 1.7 million pigs [5] are raised extensively in family backyards [6,7]. The majority of these households keep between one and four pigs of mixed breeds and the pigs have an important role as a source of meat, income and to act as a family security asset [7]. The pigs are mainly fed with kitchen waste and rice bran [6].

Pig production in Cambodia suffer from high mortality losses caused by a number of diseases including various parasitic diseases [6]. This has many reasons, often including inadequate feeding [4], insufficient veterinary and agricultural extension services [7], poor knowledge about diseases and poor access to drugs and veterinary services [6]. Two important parasitic diseases among pigs in Southeast Asia are cysticercosis and *Trichinella* spp. infection [8,9] since these are also zoonotic, i.e., diseases that can also infect humans from pigs, while the pigs themselves are seldom clinically affected. Globally 2.6–8.3 million humans are estimated to suffer from neurocysticercosis and about 28,000 deaths were attributed to cysticercosis in 2010, making it a leading cause of death from food-borne diseases [10]. The infection rate may vary significantly within and between countries, with one study showing the rate of human taeniasis varying between 0.8 and 23% and cysticercosis between 1.7 and 13% in Indonesia, but only up to 2.6% of pigs reported to have cysticercosis, while Nepal were estimated to have between 10 and 50% of humans having taeniasis and 32.5% of pigs having cysticercosis [11].

A major risk factor for porcine cysticercosis is free roaming pigs with access to human faeces [12,13,14], and a risk factor for porcine *Trichinella* spp. infection is feeding food waste containing meat to the pigs [15]. These risk factors are both common in rural small-scale pig production in Cambodia and hence increase the risks for humans to acquire these zoonotic parasitic diseases [3,6].

As part of a larger study assessing food safety risks in Cambodian meat products [16] this study aimed at understanding parasitic food safety risks. The objectives were to determine the prevalence of pigs infected with *Taenia* spp. cysticerci and with *Trichinella* spp. muscle larvae among rural pigs in four provinces in north-eastern Cambodia and to identify possible associations between prevalence and different risk factors, such as food and hygiene habits, pig management and disease knowledge among the farmers.

## 2. Materials and Methods

### 2.1. Study Area and Selection of Farms

**Figure 1:**
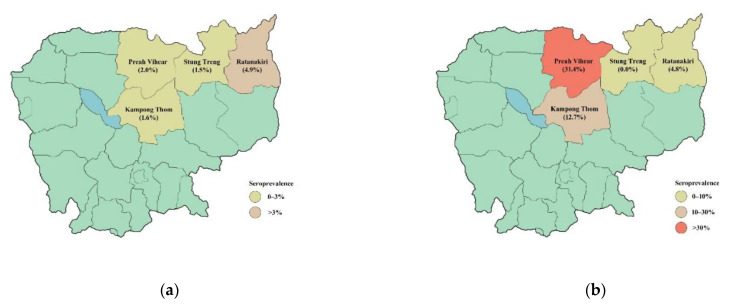
Maps illustrating the four included provinces in Cambodia and diseases in pigs. (**a**) shows the prevalence of *Trichinella* spp. infection. (**b**) shows the prevalence of cysticercosis.

This cross-sectional study was conducted in four rural provinces in northern Cambodia; Kampong Thom, Preah Vihear, Ratanakiri and Stung Treng (Figure 1), as these had been identified in expert consultations with the National Animal Health and Production Research Institute (NAHPRI) as high-risk provinces for parasitic disease with many small-scale farms keeping indigenous and free roaming pigs. Data collection was conducted in October 2019 and each province was visited for one week. Within each province, three to four districts were purposively selected by the provincial veterinarian. Districts with high numbers of free roaming pigs were prioritized, and an even geographic distribution of the districts within the province was desired. In each district, around 8 villages, where the provincial veterinarian knew there were free roaming pigs, were selected. In each village, the head of the village assisted in the selection of households based on the following criteria: the household should keep less than 10 pigs above the age of three months and the pigs should be free roaming or partly free roaming. However, due to the situation with African swine fever (ASF) in Cambodia at the time of the study, households with tethered or confined pigs were also included in the study to reach the intended number of pigs for sampling.

### 2.2. Data Collection

A structured questionnaire (Supplementary material 1) with a combination of dichotomous and multiple-choice questions was developed and targeted at the person responsible for the pigs in each household. The questionnaire contained questions regarding management of the pigs, food and hygiene habits, disease knowledge and practices related to treatment with antiparasitic drugs in the pigs. The questionnaire was developed in English and translated to Khmer. Interviews were conducted in Khmer by three veterinary students from the Royal University of Agriculture, Phnom Penh. Prior to the interview, the farmers were informed about the study per se and that their participation was voluntary and anonymous. All participants were asked for their verbal consent before the interview was conducted.

Blood samples were collected from the pigs. Sample size calculations assuming a prevalence of 20%, 5% precision and 95% confidence levels gave a sample size of 246 [17], and therefore the aim was to collect samples from 252 pigs; 63 samples from each province. A prevalence of 20% was chosen for sample size calculation, as one study in Lao had found that the prevalence of *Trichinella* spp. infection in pigs was over 14% [18], and it was possible that these extensively kept pigs would be even more exposed, and thus it was better to increase the assumed prevalence to increase the sample size and the power of the study. Cysticercosis levels have been found to vary between different countries and areas, but it was assumed Cambodia would not be as infected as the study in Nepal where more than 30% of pigs were positive for cysticercosis [11]. In each household, one to three healthy pigs were selected, preferably from different age groups. To minimize the risk of interference with maternal antibodies, which can be present for up to two months [19], and to avoid stressing piglets, no pigs younger than three months were sampled. Sampling of pregnant sows was avoided since farmers were concerned about the stress and risk of spontaneous abortion. Blood was collected from the jugular vein or from the ear vein from each pig and transferred to a vacuum serum blood collection tube which was labelled with a unique identifier code. An individual blood sample form was filled in for each sampled pig, containing information about sex, age, and breed. Samples were kept in a cooling box with ice until centrifugation was performed within 24 h of sampling. The sera were transferred into cryotubes labelled with farm and pig number and were stored in a cooling box until moved to a freezer (−18 °C) in the province. During transportation to Phnom Penh, the tubes were kept in a cooling box and were re-frozen after arrival at NAHPRI.

### 2.3. Serological Analyses

All samples were analyzed at NAHPRI through enzyme-linked immunosorbent assays (ELISA) according to instructions from the manufacturers. All samples were run in duplicates. For cysticercosis, the apDia Cysticercosis Antigen (Ag) ELISA kit (apDia bvba, Turnhout, Belgium), which uses monoclonal antibodies (IgG isotype) to detect excretory secretory products (ESP), was used for determination of viable cysticerci of *Taenia* spp. An antigen ELISA was selected as it had reportedly high sensitivity and specificity for cysticercosis. Based on the data from the manufacturer, the sensitivity could be calculated to be 96.9% for humans or animals with living cysts, and the specificity to 99.3%. The antibodies used in the assay have been produced against ESP of *T. saginata* cysticerci, and the test is only genus specific, and does not differentiate different *Taenia s*pecies.

A sample was considered positive if the antigen index was above or equal to 1.3. The antigen index of each sample was calculated by dividing the OD value of the sample by the cut-off value. The cut-off value was calculated by multiplying the mean OD value of the negative control with 3.5 according to the instructions of the manufacturer.

For *Trichinella* spp., a serological ELISA, PrioCHECK Trichinella Ab (Thermofisher Scientific, Schlieren-Zurich, Switzerland) was used to detect presence of antibodies against *Trichinella* spp. A sample was considered positive if the OD of the sample divided by the OD of the positive control exceeded the cut-off of 15% according to the instructions of the manufacturer. A study commissioned by the kit manufacturer showed that this kit has a sensitivity of 97.1–97.8% and a specificity of 99.5–99.8% [20]. The lowest estimate was used to calculate positive predictive values and true prevalence.

Test validation was performed with positive and negative controls according to the manufacturers’ instructions, and all samples were analysed in duplicates. If validation failed, the samples were reanalysed using spare ELISA plates.

### 2.4. Statistical Analysis

Data was entered into Microsoft Office Excel 2016 and statistical analysis was performed in STATA 14.2 (StataCorp, College Station, TX, USA). Descriptive statistics were calculated to define farm characteristics and pig management. Possible risk factors for seropositivity among the pigs as well as possible risk factors for infection in humans, and factors affecting human knowledge and behaviours, were further investigated. Pearson Chi2 was used for statistic between two categorical variables and Fischer’s exact test was used when Pearson Chi2 was not applicable. T-test was used for analysing age as a continuous variable. Significance level was set to *p* < 0.05.

## 3. Results

### 3.1. Description of Households

In total, 139 households were included in the study; 39 were located in Kampong Thom, 34 in Preah Vihear, 35 in Ratanakiri and 31 in Stung Treng. The majority of the respondents were females (75.4%), and the mean age was 41 (Standard Deviation (SD) 11.7) (Table 1). Most respondents (42.6%) had only attended primary school and one-third (34.6%) were uneducated.

The mean number of pigs per household was 4.2 (SD = 3.2). Most households (66.2%) kept their pigs confined in pens, while 40.3% kept them tethered, and 25.9% kept them partly confined (Table 2). In 11.5% of the households, the pigs were let to roam freely during at least some part of the year. Almost 80% of the households fed their pigs with kitchen or food waste from markets or local restaurants. Of these, 70% stated that the food waste could sometimes contain meat, and 39.0% did not cook the food waste before feeding it to the pigs. Around half of the participating households (54.7%) treated their pigs with antiparasitic drugs, but the type of drugs was not specified in the survey.

The average age of the pigs in Kampong Thom and Preah Vihear was 12.5 (SD 13.5) and 11.7 (SD 9.0) months, respectively, which was significantly higher than in Ratanakiri and Stung Treng where the sampled pigs were 6.7 (SD 6.8) and 5.4 (SD 3.9) months, respectively. Only 5% of the pigs were older than 2 years.

### 3.2. Food and Hygiene Habits

Nearly all households (98.6%) consumed pork, most commonly two to five times per week (41.9%) (Table 3). Among the pork consumers, 96.3% cooked the meat until it was brown or grey throughout before consumption. However, 20.7% sometimes consumed pork that was raw or not properly cooked. Most households (91.2%) purchased pork from the market or the mobile market (a motorbike driving around in the villages selling meat). However, one-third (33.8%) of the respondents sometimes slaughtered their own pigs, of which 89.4% used the meat for own consumption, while 25.5% sold it to others. Several responses were possible for this question, and some households reported both practices.

The respondents were shown a picture of meat with cysticercosis cysts and asked if they had ever seen similar cysts when cooking pork. Of the 28.9% that answered ‘yes’, 89.7% chose to not eat any of the meat, whereas 7.7% removed the affected parts and ate the rest, and 2.6% cooked the meat extra well before consumption (Table 4).

Most households (74.0%) had a pit latrine, while 19.7% used the bush/field as a toilet (Table 5). Only two households had a flushing toilet. In 89.1% of the families, the members always, or usually, washed their hands before eating, while in 10.9% of the families, the members never, or rarely, did this.

### 3.3. Awareness of Cysticercosis and Trichinellosis

Of the participating households, 75.5% had heard of cysticercosis, of which 76.5% claimed that they could explain what it is. The majority (98.1%) were aware that pigs could get infected, but only 22.1% knew that humans could get infected too. Of the respondents who knew that humans can get infected, 91.3% claimed that they knew how people get infected. However, out of the respondents who knew that pigs can get infected, only 22.4% claimed to know how pigs get infected. Correspondingly, 5% of the respondents had heard of trichinellosis, of which half reported to be able to explain the disease. All of the six participants answering the follow up questions were aware that pigs could get infected, but only three were aware that humans could get infected.

### 3.4. Prevalence and Risk Factors

In total, 11.2% of the sampled pigs (*n* = 242) tested positive for cysticercosis and 2.5% tested positive for *Trichinella* spp. infection (*n* = 238 due to four missing results from the ELISA) (Table 6). The true prevalence for cysticercosis (given 96.9% sensitivity and 99.3% specificity as reported by the manufacturer) was 10.9%, and for *Trichinella* spp. infection (given 97.1% sensitivity and 99.5% specificity as reported by the manufacturer) 2.1%. Pigs positive for *Taenia* spp. cysts were significantly (*p* = 0.002) older (mean 14.1 months, SD 2.9) than negative pigs (8.2 months, SD 0.6). There was no significant effect on age on seropositivity for *Trichinella* spp. infection.

Preah Vihear had a significantly higher cysticercosis prevalence compared with the other provinces (*p* < 0.001). The prevalence of cysticercosis was significantly higher in free-roaming and partly confined pigs (*p* < 0.001), while there was a significantly lower prevalence among pigs confined in pens (*p =* 0.004). For pigs that could come in contact with the toilet/stool, the prevalence of cysticercosis was significantly higher (*p* < 0.001). No association between type of toilet and cysticercosis prevalence was found. Neither was there any significant association between having seen cysts in the pork meat when preparing it and porcine cysticercosis. A significant (*p =* 0.002) association between the sex of the respondent and whether the respondent had heard of cysticercosis was found, where males were more likely to report having heard of the disease. No associations were found between knowledge of cysticercosis and whether the pigs could come in contact with stool, type of toilet, if the pigs were confined in pens or education level of the respondent. For *Trichinella* spp. infection, there was a significant association between seropositivity and feeding the pigs with food waste (*p =* 0.048). Antiparasitic treatment of pigs was significantly (*p* = 0.005) associated with lower risk for *Trichinella* spp. infection. However, no additional associations between *Trichinella* spp. seropositivity and management could be found.

Male respondents had a higher tendency to consume inadequately cooked pork meat (i.e., pink/red/raw) compared with female respondents (*p* < 0.001). The tendency was also higher among respondents in Ratanakiri (*p* < 0.001), and among respondents that reported to have heard about cysticercosis (*p* < 0.004).

## 4. Discussion

There was a significant difference in antigen prevalence of cysticercosis between the provinces, where Preah Vihear had a higher prevalence (31.4%; *p* < 0.001) compared with the other provinces. In Preah Vihear, it was not as common to keep pigs confined in pens as in the other provinces, and in almost two-thirds of the households, the pigs could come in contact with human faeces. Around 40% of the respondents in Preah Vihear had at one or more occasions seen cysts in the pork meat. Despite this, the knowledge of cysticercosis seemed to be lowest in Preah Vihear, where more than 40% of the respondents never had heard of the disease. These results confirm that management systems are of importance in preventing the disease. Absence of adequate meat inspection at the slaughterhouses, in combination with lack of knowledge of the disease in an area with presence of porcine cysticercosis, may pose a public health risk. Proper education of the veterinarians and staff responsible for the meat inspection at the slaughterhouses is important. Since home slaughter is a common practice, as shown in this study, it is also important to educate farmers on the disease. Men were significantly more likely to have heard of cysticercosis in the present study, and therefore women should initially be targeted.

The prevalence of cysticercosis was higher in this study compared to a study conducted in south-central Cambodia where the prevalence among pigs from smallholders was 7.6% [21]. The higher prevalence in the present study might be explained by the characteristics of the farms included, since high-risk areas and farms were purposely selected. In the study by Adenuga et al. [21] there was only one smallholder farm (0.9%, *n* = 115). However, porcine cysticercosis was also prevalent among confined pigs that were not supposed to have access to human faeces. In the present study, 8.4% of the confined pigs were positive for cysticercosis. This prevalence was lower compared to the prevalence of pigs that were not confined (22.2%). This could potentially be explained by environmental contamination, an explanation that has also been highlighted by Braae et al. [22]. Many of the farms had free-roaming poultry which could easily access the pig pens. Moreover, piglets were often free-roaming, and pigs often escaped the pens.

Most respondents (76%) had heard of cysticercosis, but few knew about the infection route. Similar results have been reported in a study in Tanzania, where 93% of pig keepers were aware of porcine cysticercosis and 23% knew how pigs got infected [23]. In the present study, respondents that had heard of cysticercosis were significantly more likely to consume raw or undercooked pork meat, although that is a risk factor for infection with *Taenia solium* [14,24]. This might imply that “heard of” a disease is not an adequate question to measure the knowledge level among the respondents. Additionally, many respondents might answer ’yes’ simply to provide an answer they believe to be satisfactory, so called social desirability bias [25].

The seroprevalence of porcine *Trichinella* spp. infection was only 2.5% in this study, despite targeting high risk areas. Studies conducted in neighboring countries have found the seroprevalence to be 5.6% in Vietnam [26], and ranges between 2.1% and 14.4% in different provinces in Lao PDR [18,27]. In the present study, the highest seroprevalence of *Trichinella* spp. infection within a province was in Ratanakiri (4.9%), which was significantly higher compared with the other provinces in the study. Furthermore, respondents in Ratanakiri were more likely to consume raw or undercooked pork, which is the most common infection route for humans to acquire trichinellosis [28,29]. Although we only investigated the seroprevalence among the pigs, people’s behaviours evidently affect the risk of getting infected. In that respect, Ratanakiri could be considered a province with a higher risk of human trichinellosis.

There was a considerable difference in the reported awareness of the diseases, where almost 76% had heard of cysticercosis, compared with 5% that had heard of trichinellosis. This could perhaps be explained by the fact that, in the case of cysticercosis, visible cysts can be observed in the meat which is not the case for *Trichinella* spp. infection. Information campaigns should therefore be conducted in the country to inform about the risk of consuming inadequately cooked pork meat, since all diseases are not detectable by the human eye. These campaigns should not only be targeted towards farmers, but to the general public. Most meat was reported to be purchased through informal sources, such as the mobile markets, and thus the meat does not undergo any sanitary meat inspections, which also often is lacking in official slaughterhouses [21].

Feeding kitchen waste or waste from markets and restaurants to the pigs was commonly practiced by the households. This waste could often contain meat and almost 40% reported not cooking it properly before feeding it to the pigs. Feeding inadequately cooked meat to pigs is a known risk factor for *Trichinella* spp. infection in pigs [15]. In the present study, we found an association between the practice of feeding food waste and pigs being seropositive for *Trichinella* spp. infection. Other known risk factors are exposure to rodents and wildlife [15,30]. Although no association between housing system and *Trichinella* spp. infection was found in the present study, most farms could not prevent smaller animals from entering the pig pens. However, due to the current situation with African swine fever in Cambodia at the time of the study (October 2019), many farmers had temporarily confined their pigs. In 2017, a large outbreak of human trichinellosis occurred in the Kampong Thom province in Cambodia after consumption of raw wild pig meat infected with *Trichinella papuae* [31], making possible feeding of waste from carcasses from hunted wild animals to domesticated pigs another plausible way of transmission of *Trichinella* spp. from the wild to the domestic habitat.

Treatment with antiparasitic drugs was shown to be associated with a lower risk for *Trichinella* spp. infection. However, this study did not investigate what kind of antiparasitic drugs had been used, how or when the drugs were administrated, or at what dosage, which are factors that most likely affect the treatment results. Furthermore, the seroprevalence of porcine *Trichinella* spp. infection in the studied villages was low, thereby making it uncertain whether the parasite was even prevalent among the pigs that received the antiparasitic treatment. Thus, this could be treated as a distracting factor. Moreover, it is conceivable that farmers who treat their animals with antiparasitic drugs also feed their pigs proper food, hence preventing *Trichinella* spp. infection.

Use of ELISA has limitations in diagnosing parasitic diseases, but compared to other serological methods, it is easy to use and can be performed in most laboratories, which is why it was selected for this study. Detecting antibodies is only evidence of previous exposure, but as the infection with *T. spiralis* can last more than 2 years [32] and less than 5% of the pigs in this study were older than this, it can be used to assess prevalence. The ELISA used to detect antibodies against *Trichinella* spp. had a relatively high sensitivity (97.1–97.8%) and specificity (99.5–99.8%) [20]. However, even with a specificity as high as 99.5%, the low prevalence (2.5%) found in this study means that the positive predictive value of a positive result is also low; only 83%.

For the cysticercosis antigen detection ELISA, test performance had been evaluated in a study with 31 infected animals where all the samples gave positive results indicating a high sensitivity [33]. Theoretically, an ELISA can be sensitive enough to detect one viable cysticerci [34]. However, one publication using an in-house antigen ELISA found low sensitivity and specificity in rural pigs, where many pigs only had very few cysticerci [35]. One problem with the assay used is that it is not species-specific for *Taenia* spp., which has to be taken into consideration since other species than *Taenia solium* are prevalent in Asia, e.g., *Taenia asiatica*. In Southeast Asia, *T. solium* and *T. asiatica* are both present, but the distribution of *T. asiatica* in Cambodia is unknown, perhaps because molecular methods are necessary to differentiate it from *T. saginata* which causes cysts in cattle [36,37,38]. Therefore, these results can only be interpreted as positive for *Taenia* spp. cysts, but both *T. solium* and *T. asiatica* cause taeniasis in humans and have public health importance. Given the sensitivity and specificity from the manufacturer, the positive predictive value at a prevalence of 11% was 94.5%. Another limitation of the cysticercosis assay is that it does not detect degenerated or calcified cysticerci, only viable cysticerci, and that circulating antigens are not detectable earlier than two weeks post infection, as shown in experimentally infected pigs [34]. In absence of viable cysticerci, assays that detect levels of circulating antibodies can be used to investigate exposure of the parasite [39]. However, antibodies are rarely present in cases with calcified cysts [14]. If serological tests detecting antibodies instead of antigens had been used in this study, it could have impacted the results by identifying more animals exposed to *Taenia* spp., due to presence of antibodies not being interpreted solely as ongoing infection, seeing as detectable antibodies also may derive from, e.g., passive immunity (transfer of maternal antibodies) or previous infection [40]. Furthermore, studies have shown that pigs infected with *T. solium* can suffer from behavioural changes and develop clinical signs, such as seizures, which are similar to human neurocysticercosis [41,42]. Viable cysticerci have mild or no surrounding inflammation, while degenerating cysts provoked by immune-mediated inflammation are linked to the clinical manifestations [14,43]. The methods used in this study would have been unable to detect a pig that only had degenerated cysticerci, and thereby making it impossible to know how many pigs might have been missed. However, an earlier study, which performed dissections on 18 pigs with infections confirmed with dissections, found that 3 pigs had only dead cysticerci, and no viable cysticerci at all [35]. This may imply that for every 5 pigs found with a method detecting viable cysticerci, 1 pig with degenerated cysticerci is missed. In humans, calcified cysts are associated with seizures, and considering this, infected pigs with degenerating or calcified cysts might have shown clinical signs of illness and therefore not been sampled. Meaning, that the assay in this study may have failed to detect some infected pigs, although, this is not likely to be of major importance considering the moderate prevalence found in this study.

## 5. Conclusions

In this study, the prevalence of *Taenia* spp. cyst antigens and antibodies to *Trichinella* spp. muscle larvae among pigs in rural Cambodia were 11.2% and 2.5%, respectively. The prevalence of the two infections varied significantly by province. Management systems for the pigs and pigs’ access to toilet/stool were two risk factors significantly associated with positivity for porcine cysticercosis. Seropositivity for porcine *Trichinella* spp. infection were significantly associated with feeding food waste to the pigs. Some previously demonstrated risk factors for human cysticercosis and trichinellosis were prevalent. Several different risk factors could be identified for porcine cysticercosis and *Trichinella* spp. infection. The results of this study can be used to give recommendations to improve both porcine and human health in these provinces, especially in the provinces at higher risk. Further research in other parts of Cambodia would be of interest to get a better understanding of the situation and epidemiology of these two zoonotic diseases, and should, in particular, include humans to determine the potential public health risk.

## Figures and Tables

**Table 1:** Sex, age, and education level of the respondents in the four provinces in Cambodia.

	Kampong Thom % (*n*)	Preah Vihear % (*n*)	Ratanakiri % (*n*)	Stung Treng % (*n*)	All % (*n*)
Sex of respondent (*n* = 134)					
Female	89.7 (35)	78.8 (26)	54.8 (17)	74.2 (23)	75.4 (101)
Male	10.3 (4)	21.2 (7)	45.2 (14)	25.8 (8)	24.6 (33)
Education level of respondent (*n* = 136)					
No education	48.7 (19)	29.4 (10)	43.8 (14)	12.9 (4)	34.6 (47)
Primary school	33.3 (13)	44.1 (15)	40.6 (13)	54.8 (17)	42.6 (58)
Lower Secondary	7.7 (3)	17.6 (6)	6.3 (2)	12.9 (4)	11.0 (15)
Upper Secondary	10.3 (4)	5.9 (2)	9.4 (3)	19.4 (6)	11.0 (15)
College/University	0 (0)	2.9 (1)	0 (0)	0 (0)	0.7 (1)
Mean age (SD)	40 (19–65)	38 (18–57)	42 (25–74)	45 (21–64)	41 (18–74)

**Table 2:** Housing system for the pigs in four provinces in Cambodia *.

	Kampong Thom % (*n*)	Preah Vihear % (*n*)	Ratanakiri % (*n*)	Stung Treng % (*n*)	All % (*n*)
Confined in pen	66.7 (26)	38.2 (13)	77.1 (27)	83.9 (26)	66.2 (92)
Tethered	48.7 (19)	41.2 (14)	28.6 (10)	41.9 (13)	40.3 (56)
Partly confined	5.1 (2)	70.6 (24)	22.9 (8)	6.5 (2)	25.9 (36)
Free roaming	10.3 (4)	23.5 (8)	5.7 (2)	6.5 (2)	11.5 (16)

* Several response alternatives were possible.

**Table 3:** Pork consumption habits among respondents in four provinces in Cambodia.

	Kampong Thom % (*n*)	Preah Vihear % (*n*)	Ratanakiri % (*n*)	Stung Treng % (*n*)	All % (*n*)
Pork consumption *					
Every day	5.4 (2)	29.4 (10)	26.5 (9)	9.7 (3)	17.6 (24)
2–5 times/week	21.6 (8)	41.2 (14)	44.1 (15)	64.5 (20)	41.9 (57)
Once a week	10.8 (4)	2.9 (1)	8.8 (3)	6.5 (2)	7.4 (10)
2–3 times/month	13.5 (5)	20.6 (7)	0 (0)	16.1 (5)	12.5 (17)
Once a month	48.6 (18)	5.9 (2)	20.6 (7)	3.2 (1)	20.6 (28)
Cooking level of pork **					
Cooked (brown/grey)	100 (37)	100 (34)	93.9 (31)	90.3 (28)	96.3 (130)
Cooked (red/pink)	0 (0)	0 (0)	75.8 (25)	6.5 (2)	20.0 (27)
Uncooked (raw)	0 (0)	0 (0)	0 (0)	3.2 (1)	0.7 (1)
Frequency of eating raw/red/pink ***					
Sometimes	-	-	96.0 (24)	100 (1)	96.2 (25)
Often	-	-	4.0 (1)	0 (0)	3.8 (1)

* All but two respondents (both in Kampong Thom) reported to consume pork and where asked a follow-up question about frequency of consumption. One of these did not respond to this question (*n* = 136). ** Several responses possible. *** Two of the respondents (both in Stung Treng) that reported to consume raw/red/pink pork did not answer this follow-up question (*n* = 28).

**Table 4:** Practices related to observations of cysts in pork in four provinces in Cambodia.

	Kampong Thom % (*n*)	Preah Vihear % (*n*)	Ratanakiri % (*n*)	Stung Treng % (*n*)	All % (*n*)
Have you seen cysts in the meat?					
Yes	8.1 (3)	41.2 (14)	15.2 (5)	54.8 (17)	28.9 (39)
No	91.9 (34)	58.8 (20)	84.8 (28)	45.2 (14)	71.1 (96)
If yes, what did you do?					
Did not consume	33.3 (1)	85.7 (12)	100 (5)	100 (17)	89.7 (35)
Cut away part with cysts	66.7 (2)	7.1 (1)	0 (0)	0 (0)	7.7 (3)
Cooked it extra well before consumption	0 (0)	7.1 (1)	0 (0)	0 (0)	2.6 (1)

**Table 5:** Sanitary and hygiene habits among the included households in four provinces in Cambodia.

	Kampong Thom % (*n*)	Preah Vihear % (*n*)	Ratanakiri % (*n*)	Stung Treng % (*n*)	All % (*n*)
Kind of toilet					
Flushing toilet	2.6 (1)	3.0 (1)	0 (0)	0 (0)	1.6 (2)
Pit latrine	82.1 (32)	66.7 (22)	58.6 (17)	88.5 (23)	74.0 (94)
Bush/field	2.6 (1)	30.3 (10)	41.4 (12)	7.7 (2)	19.7 (25)
Other	12.8 (5)	0 (0)	0 (0)	3.8 (1)	4.8 (6)
Can pigs come in contact with stool?					
Yes	35.9 (14)	63.6 (21)	12.5 (4)	12.9 (4)	31.9 (43)
No	64.1 (25)	36.4 (12)	87.5 (28)	87.1 (27)	68.1 (92)
Washed hands before eating					
Yes, always	20.5 (8)	58.8 (20)	90.9 (30)	80.6 (25)	60.6 (83)
Most of the times	48.7 (19)	35.3 (12)	9.1 (3)	16.1 (5)	28.5 (39)
Not so often	30.8 (12)	2.9 (1)	0 (0)	3.2 (1)	10.2 (14)
Never	0 (0)	2.9 (1)	0 (0)	0 (0)	0.7 (1)

**Table 6:** Serological results for porcine cysticercosis and *Trichinella* spp. infection in four provinces in Cambodia.

	Kampong Thom % (*n*)	Preah Vihear % (*n*)	Ratanakiri % (*n*)	Stung Treng % (*n*)	All % (*n*)
Cysticercosis					
Positive	12.7 (8)	31.4 (16)	4.8 (3)	0 (0)	11.2 (27)
Negative	87.3 (55)	68.7 (35)	95.2 (60)	100 (65)	88.8 (215)
*Trichinella* infection					
Positive	1.6 (1)	2.0 (1)	4.9 (3)	1.5 (1)	2.5 (6)
Negative	98.4 (60)	98.0 (50)	95.1 (58)	98.5 (64)	97.5 (232)

## Data Availability

Data will be made available from the corresponding author on request.

## Supplementary Materials

The following are available online at https://www.mdpi.com/article/10.3390/tropicalmed6020100/s1, Supplementary material 1: Questionnaire.

## References

[1] World Bank the World Bank in Cambodia–Overview. https://www.worldbank.org/en/country/cambodia/overview.

[2] Ly S., Sanchez Martin M.E., Phim R., Ky L., Tong K., Provo A.M., Nagpal S., Vashakmadze E.T. (2019). Cambodia Economic Update: Recent Economic Developments and Outlook.

[3] CIA East Asia/Southeast Asia: Cambodia–The World Factbook. https://www.cia.gov/library/publications/the-world-factbook/geos/cb.html.

[4] Huynh T.T.T., Aarnik A.J.A., Drucker A., Verstegen M.W.A. (2007). Pig production in Cambodia, Laos, Philippines, and Vietnam: A Review. Asian J. Agric. Dev..

[5] FAO FAOSTAT–Live Animals. http://www.fao.org/faostat/en/#data/QA.

[6] Samkol P., Borin K., Sovann S., Thorpe W., Jemaneh T. (2006). Pig systems in Southeast Asia–The case of Cambodia. Pig Systems in Asia and the Pacific: How Can Research and Development Enhance Benefits to the Poor? Proceedings of the Regional Workshop, Bangkok, Thailand, 23–24 November 2006.

[7] Sovann S., San S. (2002). Pig Production in Cambodia: Priorities for Pig Research in Southeast Asia and the Pacific to 2010.

[8] Pozio E. (2001). New patterns of Trichinella infection. Vet. Parasitol..

[9] Dorny P., Somers R., Cam T., Dang T., Nguyen V.K., Vercruysse J. (2004). Cysticercosis in Cambodia, Lao PDR and Vietnam. Southeast Asian J. Trop. Med. Public Health.

[10] Havelaar A.H., Kirk M.D., Torgerson P.R., Gibb H.J., Hald T., Lake R.J., Praet N., Bellinger D.C., de Silva N.R., Gargouri N. (2015). World Health Organization Global estimates and regional comparisons of the burden of foodborne disease in 2010. PLoS Med..

[11] Rajshekhar V., Joshi D.D., Doanh N.Q., Van De N., Xiaonong Z. (2003). Taenia solium taeniosis/cysticercosis in Asia: Epidemiology, impact and issues. Acta Trop..

[12] Pouedet M.S.R., Zoli A.P., Nguekam, Vondou L., Assana E., Speybroeck N., Berkvens D., Dorny P., Brandt J., Geerts S. (2002). Epidemiological survey of swine cysticercosis in two rural communities of West-Cameroon. Vet. Parasitol..

[13] Komba E.V.G., Kimbi E.C., Ngowi H.A., Kimera S.I., Mlangwa J.E., Lekule F.P., Sikasunge C.S., Willingham A.L., Johansen M.V., Thamsborg S.M. (2013). Prevalence of porcine cysticercosis and associated risk factors in smallholder pig production systems in Mbeya region, southern highlands of Tanzania. Vet. Parasitol..

[14] Murrell K.D., Dorny P., Flisser A., Geerts S., Kyvsgaard N.C., Mcmanus D., Nash T., Pawlowski Z. (2005). WHO/FAO/OIE Guidelines for the Surveillance, Prevention and Control of Taeniosis/Cysticercosis.

[15] Dupouy-Camet J., Murrell K., Dupouy-Camet J., Murrell K. (2007). FAO/WHO/OIE Guidelines for the Surveillance, Management, Prevention and Control of Trichinellosis.

[16] Rortana C., Nguyen-Viet H., Tum S., Unger F., Boqvist S., Dang-Xuan S., Koam S., Grace D., Osbjer K., Heng T. (2021). Prevalence of Salmonella spp. and Staphylococcus aureus in chicken meat and pork from Cambodian Markets. Pathogens.

[17] Naing L., Winn T., Rusli B. (2006). Sample size calculator for prevalence studies. Med. Stat..

[18] Holt H.R., Inthavong P., Khamlome B., Blaszak K., Keokamphe C., Somoulay V., Phongmany A., Durr P.A., Graham K., Allen J. (2016). Endemicity of zoonotic diseases in pigs and humans in lowland and upland Lao PDR: Identification of socio-cultural risk factors. PLoS Negl. Trop. Dis..

[19] Sikasunge C.S., Phiri I.K., Willingham A.L., Johansen M.V. (2010). Dynamics and longevity of maternally-acquired antibodies to Taenia solium in piglets born to naturally infected sows. Vet. J..

[20] Frey C.F., Buholzer P., Beck R., Marinculić A., Raeber A.J., Gottstein B., Schuppers M.E. (2009). Evaluation of a new commercial enzyme-linked immunosorbent assay for the detection of porcine antibodies against Trichinella spp.. J. Vet. Diagn. Investig..

[21] Adenuga A., Mateus A., Ty C., Borin K., Holl D., San S., Duggan V., Clark M., Smith G.J.D., Coker R. (2018). Seroprevalence and awareness of porcine cysticercosis across different pig production systems in south-central Cambodia. Parasite Epidemiol. Control.

[22] Braae U.C., Harrison W., Lekule F., Magnussen P., Johansen M.V. (2015). Feedstuff and poor latrines may put pigs at risk of cysticercosis–A case-control study. Vet. Parasitol..

[23] Komba E., Odiit M., Mbulamberi D.B., Chimfwembe E.C., Nantulya V.M. (1992). Multicentre evaluation of an antigen-detection ELISA for the diagnosis of Trypanosoma brucei rhodesiense sleeping sickness. Bull. World Health Organ..

[24] Ng-Nguyen D., Stevenson M.A., Breen K., Van Phan T., Nguyen V.A.T., Van Vo T., Traub R.J. (2018). The epidemiology of Taenia spp. infection and Taenia solium cysticerci exposure in humans in the Central Highlands of Vietnam. BMC Infect. Dis..

[25] Fisher R.J. (1993). Social desirability bias and the validity of indirect questioning. J. Consum. Res..

[26] Thi N.V., De N.V., Praet N., Claes L., Gabriël S., Dorny P. (2013). Seroprevalence of trichinellosis in domestic animals in northwestern Vietnam. Vet. Parasitol..

[27] Conlan J.V., Vongxay K., Khamlome B., Gomez-Morales M.A., Pozio E., Blacksell S.D., Fenwick S., Thompson R.C.A. (2014). Patterns and risks of trichinella infection in humans and pigs in Northern Laos. PLoS Negl. Trop. Dis..

[28] Gottstein B., Pozio E., Nöckler K. (2009). Epidemiology, diagnosis, treatment, and control of trichinellosis. Clin. Microbiol. Rev..

[29] Taylor M.A., Coop R., Wall R. (2007). Veterinary Parasitology.

[30] Momoh H.A., Bello M., Inabo H., Wada Y., Adole E.B., Madaiki B.D., Aregbe E.A. (2013). Prevalence and some risk factors associated with trichinellosis in backyard pig farms in Zaria, Nigeria. Trop. Anim. Health Prod..

[31] Caron Y., Bory S., Pluot M., Nheb M., Chan S., Prum S.H., Lim S.B.H., Sim M., Sengdoeurn Y., Sovann L. (2020). Human outbreak of trichinellosis caused by Trichinella papuae nematodes, Central Kampong Thom Province, Cambodia. Emerg. Infect. Dis..

[32] Pozio E., Merialdi G., Licata E., Della Casa G., Fabiani M., Amati M., Cherchi S., Ramini M., Faeti V., Interisano M. (2020). Differences in larval survival and IgG response patterns in long-lasting infections by Trichinella spiralis, Trichinella britovi and Trichinella pseudospiralis in pigs. Parasites Vectors.

[33] ApDia Cysticercosis Ag ELISA REF 650510 Manual, apDia bvba, Turnhout, Belgium, Not Dated. https://apdiagroup.com/wp-content/uploads/2020/03/650510-IFU-Cysticercosis-Ag-ELISA-960T-vs10-2018.pdf.

[34] Nguekam A., Zoli A.P., Vondou L., Pouedet S.M.R., Assana E., Dorny P., Brandt J., Losson B., Geerts S. (2003). Kinetics of circulating antigens in pigs experimentally infected with Taenia solium eggs. Vet. Parasitol..

[35] Sciutto E., Martínez J.J., Villalobos N.M., Hernández M., José M.V., Beltrán C., Rodarte F., Flores I., Bobadilla J.R., Fragoso G. (1998). Limitations of current diagnostic procedures for the diagnosis of Taenia solium cysticercosis in rural pigs. Vet. Parasitol..

[36] Eom K.S., Jeon H.K., Rim H.J. (2009). Geographical distribution of Taenia asiatica and related species. Korean J. Parasitol..

[37] Anantaphruti M.T., Yamasaki H., Nakao M., Waikagul J., Watthanakulpanich D., Nuamtanong S., Maipanich W., Pubampen S., Sanguankiat S., Muennoo C. (2007). Sympatric occurrence of Taenia solium, T. saginata, and T. asiatica, Thailand. Emerg. Infect. Dis..

[38] Ale A., Victor B., Praet N., Gabriël S., Speybroeck N., Dorny P., Devleesschauwer B. (2014). Epidemiology and genetic diversity of Taenia asiatica: A systematic review. Parasites Vectors.

[39] Sciutto E., Hernández M., García G., De Aluja A.S., Villalobos A.N.M., Rodarte L.F., Parkhouse M., Harrison L. (1998). Diagnosis of porcine cysticercosis: A comparative study of serological tests for detection of circulating antibody and viable parasites. Vet. Parasitol..

[40] Gavidia C.M., Verastegui M.R., Garcia H.H., Lopez-Urbina T., Tsang V.C.W., Pan W., Gilman R.H., Gonzalez A.E., Rodriguez S., Gomez L. (2013). Relationship between serum antibodies and taenia solium larvae burden in pigs raised in field conditions. PLoS Negl. Trop. Dis..

[41] Trevisan C., Mkupasi E.M., Ngowi H.A., Forkman B., Johansen M.V. (2016). Severe seizures in pigs naturally infected with Taenia solium in Tanzania. Vet. Parasitol..

[42] Trevisan C., Johansen M.V., Mkupasi E.M., Ngowi H.A., Forkman B. (2017). Disease behaviours of sows naturally infected with Taenia solium in Tanzania. Vet. Parasitol..

[43] García H.H., Gonzalez A.E., Evans C.A.W., Gilman R.H. (2003). Taenia solium cysticercosis. Lancet.

