# Supplementary material for: Low Prevalence of Cysticercosis and Trichinella Infection in Pigs in Rural Cambodia
Source: Trop Med Infect Dis. 2021 Jun 11;6(2):100. doi: 10.3390/tropicalmed6020100 (PMC8293377; doi:10.3390/tropicalmed6020100)
Supplement: Supplementary file 1 [file tropicalmed-06-00100-s001.zip › tropicalmed-1224148-supplementary.pdf]

Tropical Medicine and Infectious Disease

MDPI

# The Supplementary of Low Prevalence of Cysticercosis and *Trichinella* Infection in Pigs in Rural Cambodia

**Questionnaire/**

◯ Used for questions with only one possible answer/

☐ Used for questions with more than one possible answers/

Farm no/ : ……….

| No | *A. Household demographics/* | | | |
|---|---|---|---|---|
| **A1** | Geographic location | Province/ : ………. | | |
| | | District/ : ………. | | |
| | | Commune/ : ………. | | |
| | | Village/ : ………. | | |
| **A2** | Sex of respondent | Female / | ◯ | |
| | | Male/ | ◯ | |
| **A3** | Age of respondent/ | ………. years/ | | |
| **A4** | Highest level of education of respondent/ | College/University / | ◯ | |
| | | Upper secondary school/ | ◯ | |
| | | Lower secondary school/ | ◯ | |
| | | Primary school/ | ◯ | |
| | | No education/ | ◯ | |

| | | | | |
|---|---|---|---|---|
| **A5** | Number of people living in the household | Adults/ : (15-60 years): ………. | | |
| | | Children/ (< 15 years): ………. | | |
| | | Elderly/ (> 60 years): ………. | | |
| ***No*** | ***B. Food and hygiene habits/*** / | | | |
| **B1** | Do you eat pork meat in the family? ? | Yes / | ○ | |
| | | No/ | ○ | **Go to B8** |
| **B2** | If yes, how often? , | Every day / | ○ | |
| | | 2-5 times/week/ – / | ○ | |
| | | Once a week/ | ○ | |
| | | 2-3 times/month/ – / | ○ | |
| | | Once a month/ | ○ | |
| | | Other: ………. | ○ | |
| **B3** | How well cooked is the pork meat that you eat? ? | Uncooked (raw) / | □ | |
| | | Cooked (red/pink in the middle) | □ | **Go to B5** |
| | | Cooked (brown/grey throughout) / | □ | |
| **B4** | If uncooked, how often do you eat uncooked meat? ? | Always / | ○ | |
| | | Often/ | ○ | |
| | | Sometimes/ | ○ | |

| | | | | |
|---|---|---|---|---|
| **B5** | From where do you get the pork meat that your family consumes? | Own pigs/ | ☐ | |
| | | Neighbors' pigs/ | ☐ | |
| | | Market/ | ☐ | |
| | | Other/ : ………. | ☐ | |
| **B6** | Have you ever seen cysts in the pork meat when preparing it? *(Picture)* ( | Yes / – | ◯ | |
| | | No / | ◯ | **Go to B8** |
| **B7** | If yes, what do you do with the meat? | Eat it like normal meat/ | ◯ | |
| | | Cook it extra well / | ◯ | |
| | | Cut off the bad part and eat the rest | ◯ | |
| | | Don't eat any of it/ | ◯ | |
| **B8** | What kind of toilet do you have? | Flushing toilet connected to sewer | ◯ | |
| | | Pit latrine/ | ◯ | |
| | | Bucket toilet/ | ◯ | |
| | | Bush/field toilet/ | ◯ | |
| | | Don't have one/ | ◯ | |
| | | Other/ : ………. | ◯ | |
| **B9** | *Observational question:* Can the pigs come in contact with the toilet/stool? | Yes / / | ◯ | |

| | | | | |
|---|---|---|---|---|
| | ? | No/ | ◯ | |
| | | Most of the times/ | ◯ | |
| | | Not so often/ | ◯ | |
| | | No, never/ | ◯ | |
| **B10** | Do you and your family wash your hands before eating? ? | Yes, always/ | ◯ | |
| | | Most of the times/ | ◯ | |
| | | Not so often/ | ◯ | |
| | | No, never/ | ◯ | |
| ***No*** | ***C. Farm details/*** | | | |
| **C1** | Number of pigs (at time of visit) ( | Piglets/ (< 1 month): ………. | | |
| | | Growers/ (1-3 months): ………. | | |
| | | Fatteners/ (> 3 months): ………. | | |
| | | Breeding sows/ : ………. | | |
| | | Breeding boars/ : ………. | | |
| **C2** | Pig breed(s) on the farm | Indigenous pigs/ | ☐ | |
| | | Commercial pigs/ | ☐ | |
| **C3** | Housing system for the pigs | Tethered/ / | ☐ | |
| | | Confined in pens/ | ☐ | |

| | | | | |
|---|---|---|---|---|
| | | Partly confined/ | ☐ | |
| | | Free roaming/ | ☐ | **Go to C6** |
| **C4** | Which group(s) of pigs are tethered/confined? / | Piglets/ (< 1 month) | ☐ | |
| | | Growers/ (1-3 months) | ☐ | |
| | | Fatteners/ (> 3 months) | ☐ | |
| | | Breeding sows/ | ☐ | |
| | | Breeding boars/ | ☐ | |
| **C5** | If partly confined, what time of the day are the pigs kept confined? | Day time/ | ☐ | |
| | | Dusk/ | ☐ | |
| | | Night time/ | ☐ | |
| | | Other/ : ………. | ☐ | |
| **C6** | If partly confined/free roaming, during which season do the pigs roam free? ? | Dry season/ | ◯ | |
| | | Rainy season/ | ◯ | |
| | | Both/ | ◯ | |
| **C7** | Do you feed your pigs with kitchen waste or other food waste from markets/restaurants? | Yes/ / | ◯ | |
| | | Sometimes / | ◯ | |

| | | | | |
|---|---|---|---|---|
| | ? | No/ | ◯ | **Go to D1** |
| **C8** | If yes, does it ever contain meat? | Don't know / | ◯ | |
| | | Yes/ / | ◯ | |
| | | No/ | ◯ | **Go to D1** |
| **C9** | Do you cook the food waste before feeding it to the pigs? ? | Yes/ / | ◯ | |
| | | Sometimes / | ◯ | |
| | | No/ | ◯ | |

| *No* | *D. Disease knowledge and vaccination/* | | | |
|---|---|---|---|---|
| **D1** | Have you heard about Trichinellosis? ? | Yes / / | ◯ | |
| | | No/ | ◯ | **Go to D7** |
| **D2** | Can you explain what it is? | Yes/ / : ………. | ◯ | |
| | | No/ | ◯ | |
| **D3** | Who can get infected? *Don't read options out loud* | Humans/ | ☐ | |
| | | Pigs/ | ☐ | |
| | | Other animals/ : ………. | ☐ | |
| | *If answering "humans":* | Yes/ / : ………. | ◯ | |

| | | | | |
|---|---|---|---|---|
| **D4** | Do you know how people get infected?<br>? | No/ | ○ | |
| **D5** | Has anyone in your family or anyone else you know had Trichinellosis?<br>? | Yes, someone in the family | ☐ | |
| | | Yes, someone else | ☐ | |
| | | No/ | ○ | |
| **D6** | *If answering "pigs":*<br>Do you know how pigs get infected?<br>? | Yes/ / : ………. | ○ | |
| | | No/ | ○ | |
| **D7** | Have you heard about Cysticercosis?<br>( ) ? | Yes/ / : ………. | ○ | |
| | | No/ | ○ | **Go to D13** |
| **D8** | Can you explain what it is? | Yes/ / : ………. | ○ | |
| | | No/ | ○ | |
| **D9** | Who can get infected? *Don't read options out loud*<br>( !!!) | Humans/ | ☐ | |
| | | Pigs/ | ☐ | |
| | | Other animals/ : ………. | ☐ | |
| **D10** | *If answering "humans":* | Yes/ / : ………. | ○ | |

| | | | | |
|---|---|---|---|---|
| | Do you know how people get infected?<br><br>? | No/ | ◯ | |
| D11 | Has anyone in your family or anyone else you know had Cysticercosis?<br><br>( ) ? | Yes, someone in the family | ☐ | |
| | | Yes, someone else | ☐ | |
| | | No/ | ◯ | |
| D12 | *If answering "pigs":*<br><br>Do you know how pigs get infected?<br><br>? | Yes/ / : ………. | ◯ | |
| | | No/ | ◯ | |
| D13 | Do you treat the pigs with antiparasitic medicines?<br><br>? | Yes/ / : ………. | ◯ | |
| | | No/ | ◯ | **Go To D15** |
| | | Don't know/ | ◯ | |
| D14 | If yes, how often?<br><br>? | Every week/ | ◯ | |
| | | Every month/ | ◯ | |
| | | Every year/ | ◯ | |
| | | Other/ : ………. | ◯ | |
| ***No*** | ***G. Animal movement/*** | | | |
| **G8** | Do you slaughter pigs at home? | Yes/ / : ………. | ◯ | |

| | | | | |
|---|---|---|---|---|
| | | No/ | ◯ | **Go to G10** |
| **G9** | If yes, what do you do with the meat? | Own consumption/ | ☐ | |
| | | Sell to others/ | ☐ | |
| | | Other: ………. | ☐ | |

Blood sample form

Farm no/ : ………. (*Example: F1*) Pig no/ : ………. (*Example: P1*)

| Sex/ | | Age/ | Breed/ | |
|---|---|---|---|---|
| Female | ◯ | ………. months *or* ………. years | Indigenous | ◯ |
| Male | ◯ | | Commercial | ◯ |
